# Applications of Conductive Polymer Hydrogels for Supercapacitor, Solar Cell, and Energy Conversion

**DOI:** 10.3390/gels11090741

**Published:** 2025-09-15

**Authors:** Sabuj Chandra Sutradhar, Md. Shahriar Ahmed, Mohammad Afsar Uddin, Ye-Chan Oh, Junwoo Park, Kyung-Wan Nam, Mobinul Islam

**Affiliations:** 1 Department of Energy Materials Science and Engineering, Konkuk University, Chungju-si 27478, Republic of Korea; chandra@kku.ac.kr; 2 Department of Energy and Materials Engineering, Dongguk University-Seoul, Seoul 04620, Republic of Korea; shahriar.emcl@dgu.ac.kr (M.S.A.);; 3 Instituto de Ciencia de Materiales de Madrid, CSIC, Cantoblanco, 28049 Madrid, Spain; 4 Department of Advanced Battery Convergence Engineering, Dongguk University, Seoul 04620, Republic of Korea

**Keywords:** hydrogels, energy storage, energy conversion, polymer electrolytes, fuel cells, supercapacitors

## Abstract

Hydrogels are rapidly emerging as a versatile and promising platform for advancing energy storage and conversion technologies. Their intrinsic properties—such as high water content, excellent ionic conductivity, and inherent mechanical flexibility—position them as key materials for a wide range of applications, including supercapacitors, flexible membranes, and components in fuel cells and solar cells. Despite significant progress, challenges remain in enhancing their mechanical durability, developing scalable fabrication methods, and ensuring environmental sustainability. Recent breakthroughs in composite hydrogel systems, innovative manufacturing techniques such as 3D printing, and self-healing strategies are driving substantial improvements in device performance and operational lifespan. Emphasizing the importance of interdisciplinary approaches and innovative material design, this review highlights the transformative potential of hydrogel-based energy systems in shaping a sustainable and flexible energy future. The advancements discussed herein have promising implications for the development of high-performance, environmentally friendly, and adaptable energy devices capable of meeting the demands of next-generation applications.

## 1. Introduction

**Scheme 1:**
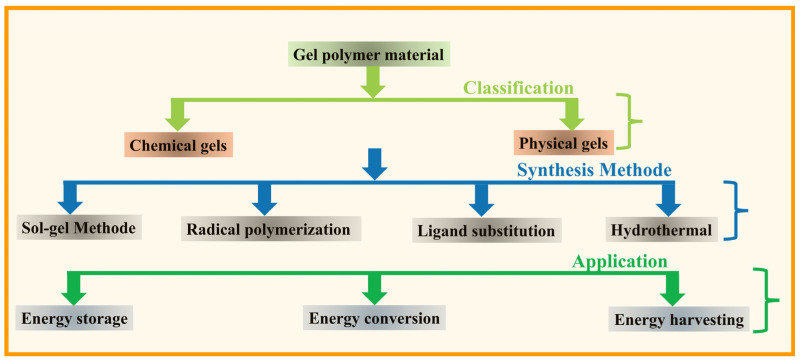
Advances in hydrogel materials: Classification, fabrication techniques, and energy storage applications.

Hydrogels have emerged as a versatile and highly promising class of functional materials for advanced energy storage and conversion systems, owing to their distinctive physicochemical attributes. These include a high water content, facilitating facile ionic transport; exceptional ionic conductivity; tunable mechanical properties enabling flexibility and compliance; and inherent biocompatibility [1,2,3,4,5]. While hydrogels are primarily recognized as highly flexible, gel-like materials capable of acting as electrolytes or separators in energy devices, some hydrogels also possess conductive properties. When they exhibit electrical conductivity, these hydrogels may be referred to as conducting polymers or polyelectrolytes. Conductive hydrogels are a distinct subclass that combine the desirable mechanical and structural features of traditional hydrogels with electrical conductivity similar to that of conducting polymers and polyelectrolytes. Structurally, hydrogels are three-dimensional, cross-linked polymer networks capable of retaining substantial quantities of water—often several times their dry weight—thus mimicking biological tissues in their softness and deformability. The high ionic mobility within hydrogel matrices enables efficient charge transfer, which is crucial for driving electrochemical reactions in devices such as supercapacitors, lithium-ion batteries, fuel cells, and photovoltaic cells [6,7]. The increasing demand for portable, wearable, and flexible electronic devices has catalyzed research efforts to integrate hydrogels into energy systems, leveraging their intrinsic properties to achieve high performance, mechanical durability, and safety. In supercapacitors, hydrogels transcend their conventional role as passive electrolytes; they function as bifunctional components that serve as electrolytic media, electrode supports, or composite electrolyte–electrode architectures. This integration facilitates the development of solid-like, highly conductive platforms capable of supporting rapid charge–discharge cycles under mechanical deformation, including stretching, bending, and twisting—attributes essential for flexible and wearable electronics [8,9,10]. Such features allow for the fabrication of robust, safe, and lightweight energy storage devices that retain electrochemical performance under dynamic operational conditions. Moreover, the intrinsic simplicity and reliability of hydrogel synthesis confer significant advantages over other complex energy materials, fostering scalable manufacturing processes [11]. Their straightforward classification, combined with adaptable chemistry, facilitates the tailoring of properties for targeted applications [12]. As summarized in Scheme 1, a comprehensive overview of hydrogel classifications, synthesis methodologies, and functional applications underscores their potential as a flexible, durable, and environmentally benign platform for next-generation energy storage and conversion technologies.

Further, the versatility of hydrogels is demonstrated through their ability to incorporate various nanomaterials—such as graphene, MXenes, or conducting polymers like polyaniline (PANI)—forming hybrid nanocomposites that significantly enhance electrochemical performance [13,14]. For example, integrating graphene oxide or conducting polymers such as polyaniline (PANI) into hydrogel matrices yields composite structures exhibiting increased specific capacitance, improved charge–discharge stability, and superior mechanical robustness. These modifications address the common trade-offs observed in traditional electrode materials, such as brittleness or limited ionic permeability, thereby enabling the development of next-generation supercapacitors that are lightweight, flexible, and suitable for real-world applications.

In addition to energy storage devices, hydrogels are increasingly being investigated in energy conversion systems, where they can substantially improve device performance and design flexibility. For instance, in dye-sensitized solar cells (DSSCs), hydrogel-based electrolytes provide high ionic conductivity while offering mechanical adaptability for fabrication of bendable or wearable photovoltaic devices [15]. Such hydrogel electrolytes facilitate effective dye molecule regeneration and electron transfer, contributing to improved power conversion efficiencies. Similarly, in fuel cell technology, hydrogel-based proton exchange membranes serve as environmentally friendly, cost-effective alternatives to Nafion membranes, offering comparable or enhanced proton conductivity, mechanical durability, and flexibility [16,17,18,19]. Their ability to be doped with acids or other functional molecules further allows fine-tuning of their electrochemical properties to optimize device efficiency and operational stability. The overall advantage of hydrogel use in energy systems extends beyond just performance improvement; these materials are inherently environmentally benign, sustainable, and capable of biodegradation, addressing growing concerns over the ecological impact of energy device components [20,21,22]. Moreover, advances in hydrogel chemistry such as the development of stimuli-responsive hydrogels offer opportunities for smart energy devices that can adapt to changing environmental conditions or operational states, increasing their utility and lifespan. The integration of nanomaterials further enhances these properties, paving the way for multifunctional hydrogel composites with targeted functionalities such as self-healing, enhanced conductivity, or environmental sensing capabilities.

Self-healing hydrogels are innovative materials capable of autonomously repairing themselves after damage, which significantly enhances their durability and lifespan. In the context of advanced hydrogel materials for supercapacitors, solar cells, and energy conversion applications, self-healing hydrogels offer several advantages. They can maintain electrical conductivity and mechanical integrity despite repeated mechanical stress or damages, ensuring consistent performance. These hydrogels typically achieve self-healing through dynamic chemical bonds, reversible physical interactions, or a combination of both, allowing them to restore their structure and properties automatically. Their ability to recover quickly and repeatedly makes them highly suitable for flexible, long-lasting energy devices, where reliability and resilience are crucial. Incorporating self-healing capabilities in hydrogels is, thus, a promising strategy to improve the efficiency, durability, and cost-effectiveness of next-generation energy storage and conversion systems.

**Figure 1:**
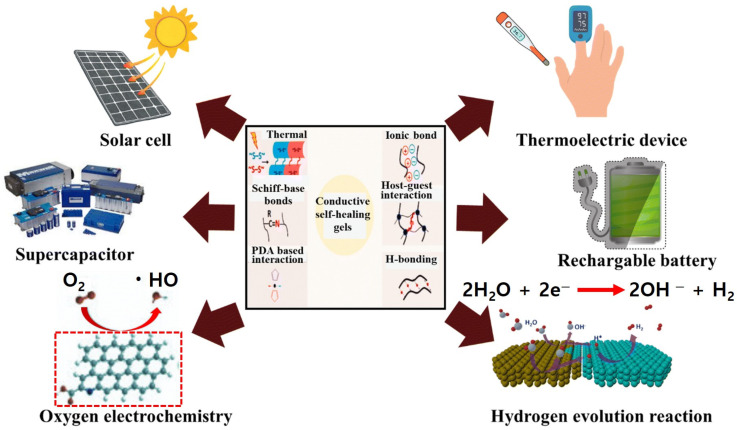
Applications of conductive self-healing gels in energy conversion and electrochemical devices, reproduced from Ref. [24] with permission from the Royal Society of Chemistry 2025.

As the field develops, a concerted effort to understand the fundamental interactions between hydrogel matrices, nanomaterials, and electrochemical interfaces will be crucial for designing optimized energy devices. Ongoing research continues to focus on improving aspects such as biocompatibility, mechanical durability, and electrochemical performance, further solidifying hydrogels’ role in flexible, sustainable, and high-performance energy systems. Their potential extends beyond simple electrolytes to include active electrodes and integrated device architectures, effectively bridging soft, biomimetic materials with high-end energy technologies. The convergence of nanotechnology, materials science, and electrochemistry in hydrogel research promises revolutionary breakthroughs for miniaturized, lightweight, and environmentally friendly energy devices suitable for next-generation portable, wearable, biomedical, and renewable energy applications [23,24]. A detailed understanding of the structure–property relationships of hydrogel-based materials will be pivotal in unlocking their full potential, especially for addressing the global pressing need for sustainable, reliable, and adaptable energy sources. Figure 1 illustrates the application of conductive self-healing gels in the realm of energy and electrochemical devices. These gels play significant roles in solar cells, thermoelectric devices, rechargeable batteries, supercapacitors, hydrogen evolution reactions, and oxygen electrochemistry.

Recent advances in hydrogel materials have highlighted the crucial role of hydration in determining ion mobility, which directly influences the performance of energy storage and conversion devices such as supercapacitors and solar cells. Increased hydration levels enhance ion mobility by expanding the polymer network and improving ionic conduction pathways, resulting in higher electrical conductivity and better device efficiency [25,26]. Hydration-induced swelling creates more free volume within the hydrogel matrix, facilitating faster ion transport essential for high-capacity supercapacitors and efficient electrochemical processes in solar energy conversion systems [27]. Conversely, excessive hydration can lead to ion dilution and reduced electrochemical gradients, potentially impairing device performance [28]. Therefore, controlling and optimizing the degree of hydrogel materials is vital for advancing their application in energy storage and conversion technologies.

## 2. Applications of Hydrogel-Derived Materials for Supercapacitors

### 2.1. Hydrogels in Supercapacitors

Hydrogels are primarily employed as electrolyte media within the supercapacitor. Their highly conductive, flexible, and ion-permeable nature makes them ideal for facilitating efficient ion transport between the electrodes during charge and discharge cycles [29]. This means that hydrogels help maintain high ionic conductivity while allowing the device to be flexible and mechanically robust. In addition to their role as electrolytes, hydrogels are also sometimes used as membranes within the supercapacitor structure. In this configuration, they act as separators that keep the electrodes apart while still permitting rapid ion movement [30]. This function not only helps prevent short circuits but also enhances the mechanical stability and flexibility of the device, which is particularly advantageous for flexible or wearable energy storage systems. Their use as electrolytes supports the fundamental charge transfer processes, while their application as membranes contributes to device durability and mechanical flexibility.

**Figure 2:**
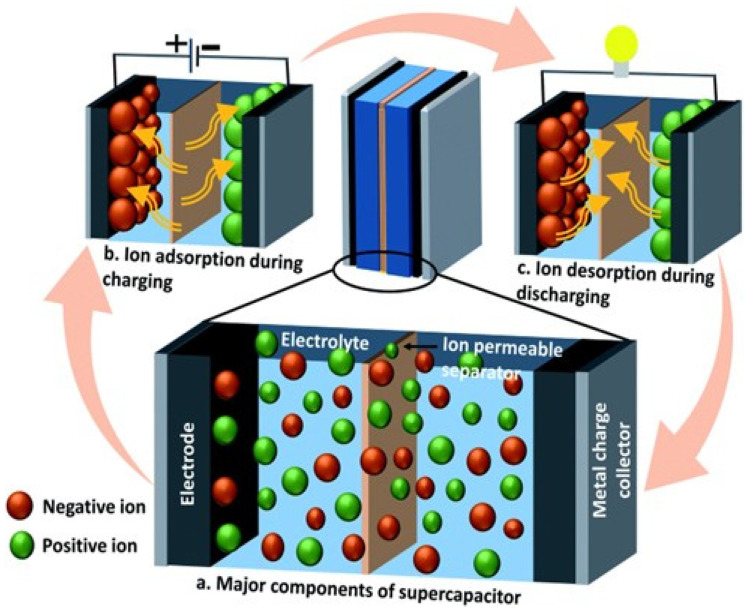
Schematic representation of an electric double-layer capacitor (EDLC), illustrating (**a**) the key components including electrodes, electrolyte, separator, and current collectors; and the operational mechanism during (**b**) charging, where ions are adsorbed at the electrode surface, and (**c**) discharging, involving ion desorption, reproduced from Ref. [31] with permission from the Royal Society of Chemistry 2021.

Supercapacitors, also known as electrochemical capacitors, are gaining prominence in energy storage due to their high power density (>10,000 W kg^−1^), long cycle life (>100,000 cycles), rapid charge/discharge capability, and excellent reversibility. These attributes make SCs ideal for applications in portable and wearable electronics. However, conventional SCs typically rely on slurry-cast electrodes composed of active materials, binders, and conductive additives, which introduce structural inefficiencies and limit mechanical adaptability (Figure 2) [31]. In the context of hydrogel applications, hydrogels act as effective electrolyte media in the supercapacitor setup. They facilitate ion movement by providing a conductive and flexible environment for charge separation and transport. During the charging process (Figure 2b), hydrogels efficiently allow cations to migrate toward the negative electrode and anions toward the positive electrode. This ion separation enhances double layer capacitance by stabilizing ion movement and accumulation at the electrode–electrolyte interface. During the discharging process (Figure 2c), hydrogels support the reverse ion movement, enabling a rapid and reliable release of stored energy. Their structure maintains mechanical integrity, supporting repeated charge–discharge cycles and contributing to the overall stability and performance of the supercapacitor. The slurry casting process results in inactive “dead volume” and exfoliation of active materials, reducing both volumetric and gravimetric capacitance. Moreover, the rigidity of traditional electrode structures hinders their integration into flexible and stretchable devices. These limitations pose significant challenges for next-generation electronics that demand both high performance and mechanical compliance. Hydrogels have emerged as promising candidates to address these challenges. Graphene-based and conductive polymer-based hydrogels, such as polypyrrole (PPy) and polyaniline (PANI), offer high electrical conductivity (e.g., PPy hydrogels: 7.8 S cm^−1^) [32], large theoretical surface area (e.g., graphene hydrogel: 2630 m^2^ g^−1^) [33], and tunable porous architectures. Synthetic hydrogels like PEO, PVA, PAAK, and PAAm, when combined with electrolytes such as KOH, H_2_SO_4_, or H_3_PO_4_, serve as efficient ion-conducting separators [34]. Their 3D polymer networks facilitate high ionic conductivity and enable multifunctional properties including stretchability, self-healing, antifreezing, and mechanochromism [35,36,37,38,39,40].

SCs operate via two primary mechanisms: electrical double-layer capacitance (EDLC) and pseudocapacitance. Graphene hydrogels are typical EDLC materials, leveraging electrostatic interactions at the electrode–electrolyte interface. In contrast, PANI and PPy hydrogels exhibit pseudocapacitive behavior through redox reactions, offering high theoretical specific capacitances of 2000 and 620 F g^−1^, respectively. Despite their high capacitance, polymer hydrogels often suffer from poor cycling stability and rate capability due to inefficient charge transfer and structural degradation. To overcome these limitations, hybrid hydrogels combining conductive polymers with graphene have been developed. These hybrids integrate the high capacitance of pseudocapacitive materials with the mechanical strength and reversibility of EDLCs, resulting in flexible, high-performance supercapacitor electrodes.

#### 2.1.1. Hydrogel-Derived Electrodes

Conductive hydrogels, such as those based on polyaniline (PANI) [41], polypyrrole (PPy) [42], poly(3,4-ethylenedioxythiophene):poly(styrene sulfonate) (PEDOT:PSS) [43], graphene hydrogels [44], and their hybrid composites [45], have demonstrated significant potential as directly applicable active or self-supported electrodes for flexible energy storage devices. For instance, PPy hydrogels synthesized via interfacial polymerization exhibit tunable microstructures, which can be controlled by adjusting the phytic acid-to-pyrrole ratio during fabrication [42]. When grown on carbon cloth substrates and combined with a PVA-H_2_SO_4_ electrolyte, these hydrogels achieved a specific capacitance of 380 F g^−1^ and showed excellent cycling stability. PEDOT:PSS hydrogels, prepared through mild hydrothermal treatment, demonstrated tunable electrical conductivities within the range of 46 to 880 S m^−1^, and could be readily fabricated into diverse geometries—including films, fibers, and columns—facilitating the development of various flexible supercapacitor configurations [42].

**Figure 3:**
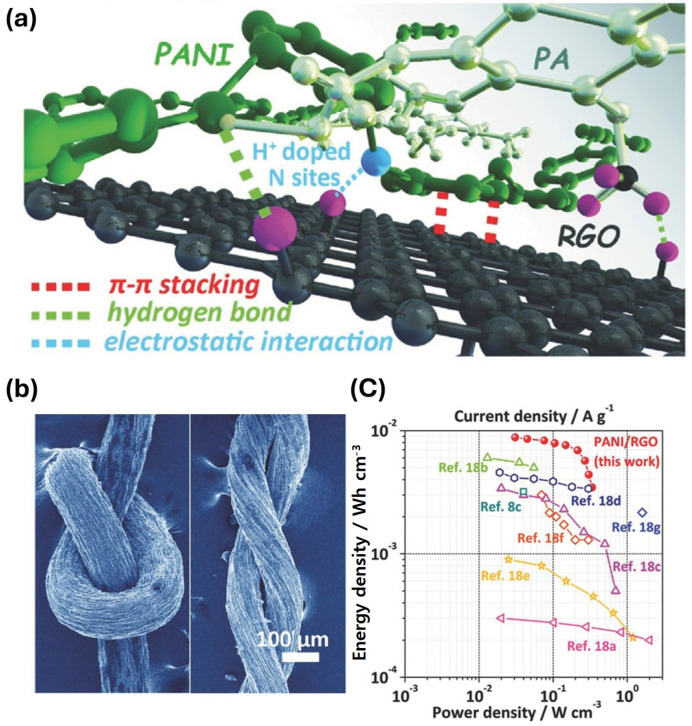
(**a**) Schematic showing the interaction among PANI, PA, and rGO components; (**b**) morphological flexibility of PANI/rGO fibers in knotted and twisted configurations; (**c**) Ragone plot comparing energy and power densities of all-gel-state full cells with other fibrous supercapacitor systems, reproduced with permission from ref. [44] Wiley & Sons 2018.

Graphene hydrogels, characterized by their three-dimensional porous networks, effectively overcome the stacking limitations caused by π–π interactions in conventional graphene sheets. These structures offer high electrical conductivity coupled with enhanced mechanical strength. Functionalization with hydroquinone further improves their electrochemical stability, with reports indicating a retention of 91.6% capacitance after 10,000 charge–discharge cycles at a current density of 10 A g^−1^ [44]. Despite these advantages, graphene hydrogels primarily operate via electrical double-layer capacitance (EDLC), which typically results in lower specific capacitance compared to pseudocapacitive polymer hydrogels. To address this limitation, hybrid hydrogels combining conducting polymers with graphene-based materials—such as PANI/rGO composites—have been developed. These hybrids utilize electrostatic interactions, hydrogen bonding, and π–π stacking to form fibrous, robust structures with excellent mechanical integrity (Figure 3a) [44].

Remarkably, a single PANI/rGO fiber with a diameter of approximately 100 μm demonstrated a tensile strength of 140 MPa at 31% strain—three times higher than RGO or PANI alone—and exhibited the ability to be knotted or twisted without fracturing (Figure 3b) [44]. An all-hydrogel-based supercapacitor employing two PANI/rGO fibers and a PVA-H_2_SO_4_ electrolyte was capable of enduring 40% tensile strain while delivering a volumetric energy density of 8.80 mWh cm^−3^ at a power density of 30.77 mW cm^−3^, surpassing many other fiber-shaped supercapacitors in performance (Figure 3c) [44].

#### 2.1.2. Hydrogel Electrolytes

Hydrogel electrolytes have become increasingly important in the development of flexible supercapacitors due to their high ionic conductivity, mechanical flexibility, and multifunctional capabilities. Unlike conventional liquid electrolytes, hydrogels can be tailored to suit the specific requirements of wearable and deformable energy storage devices, offering advantages such as shape adaptability and improved safety [45]. However, traditional hydrogel electrolytes—particularly those based on polyvinyl alcohol (PVA)—face challenges related to water retention and mechanical durability. The relatively low density of hydrophilic side chains in PVA results in weaker interactions with water molecules, leading to rapid dehydration and increased internal resistance during operation, which negatively affects long-term electrochemical stability, especially under mechanical deformation or environmental stress [46]. Hydrogels are composed of elastic, interconnected polymer networks that can absorb and hold large amounts of water—up to about 2000 times their own weight—providing a high water content that is critical for ionic conduction. Functional groups such as hydroxyl, carboxyl, sulfonic, and amino groups contribute to water retention and improve ionic mobility, thereby enhancing electrochemical performance [47]. Compared to liquid electrolytes, hydrogels exhibit superior stretchability, self-healing properties, and adjustable mechanical strength, making them highly suitable for use in flexible supercapacitors.

**Figure 4:**
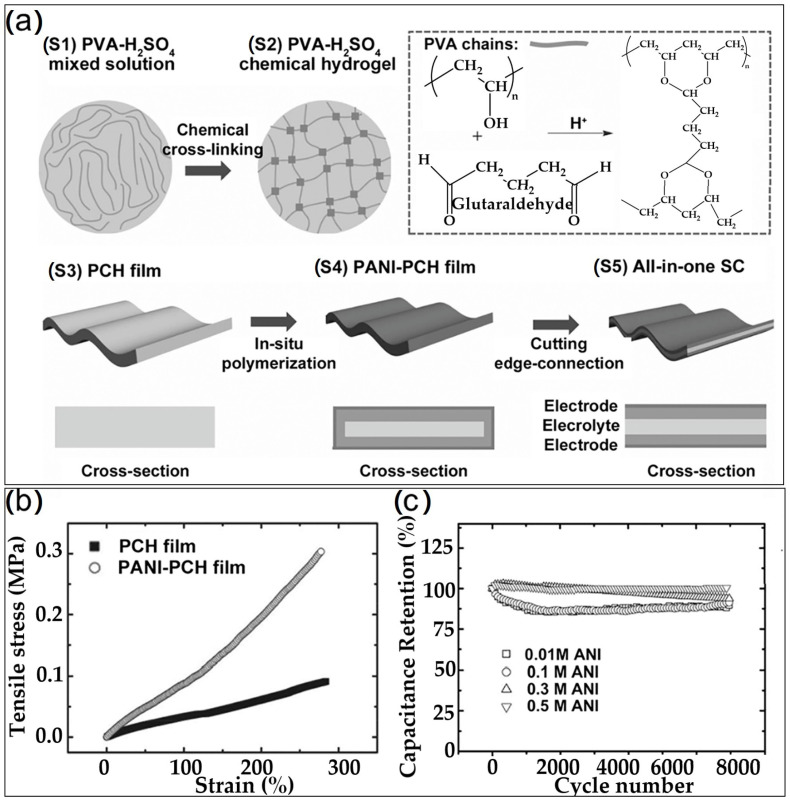
Fabrication and mechanical–electrochemical characterization of an all-in-one supercapacitor based on PANI-coated hydrogel film. (**a**) Schematic illustration of polyaniline (PANI) synthesized in situ on a glutaraldehyde-crosslinked poly (vinyl alcohol)-sulfuric acid (PVA-H_2_SO_4_) hydrogel film, forming the PANI-PCH composite. (**b**) Mechanical performance of the PANI-PCH film demonstrated through tensile stress–strain analysis. (**c**) Capacitance retention behavior of the assembled supercapacitors as a function of varying PANI layer thicknesses, reproduced with permission from ref. [48]. Wiley & Sons 2015.

To improve their mechanical stability and electrochemical efficiency, PVA hydrogels are often chemically crosslinked with glutaraldehyde (GA). This crosslinking process involves the formation of acetal bonds between hydroxyl groups on PVA and aldehyde groups in GA under acidic conditions, which greatly increases the elasticity and mechanical strength of the hydrogel network [48,49]. This modification helps overcome durability issues and enhances the overall performance of hydrogel electrolytes in flexible energy storage applications (Figure 4a).

**Figure 5:**
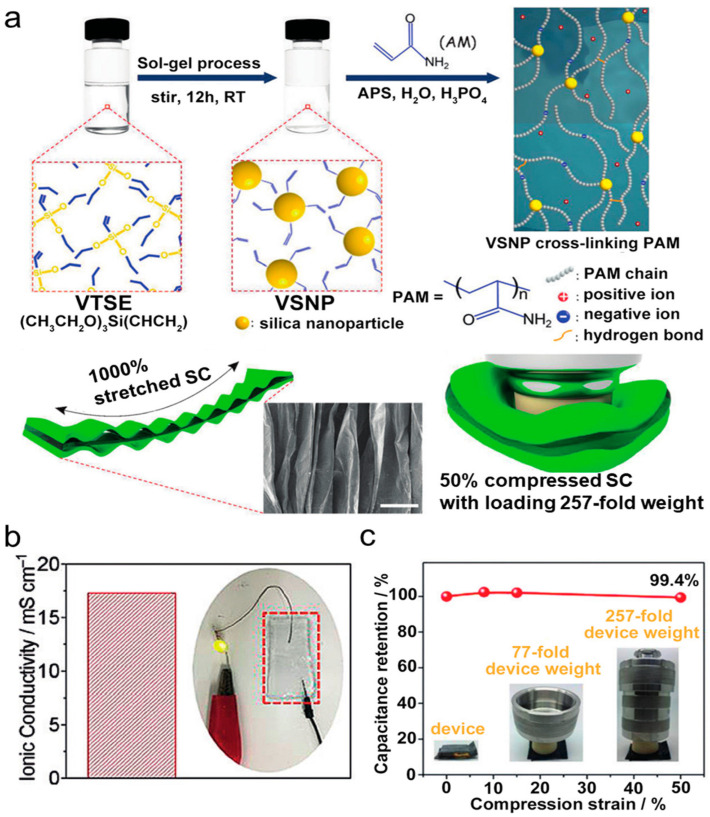
Design and functional assessment of a stretchable and compressible hydrogel-based supercapacitor. (**a**) Illustration of the synthesis process for the VSNPs-PAAm hydrogel electrolyte, engineered for enhanced mechanical adaptability. (**b**) Ionic conductivity characteristics of the hydrogel, highlighting its suitability for flexible energy storage applications. (**c**) Capacitance retention of supercapacitors utilizing polypyrrole (PPy)-electrodeposited carbon nanotube (CNT) paper electrodes and VSNPs-PAAm hydrogel electrolyte under varying compressive strains; inset images depict the device under different heavy load conditions, reproduced with permission from ref. [34], Wiley & Sons 2017.

GA-crosslinked PVA films demonstrate remarkable mechanical and electrochemical properties, with stretchability reaching up to 300%, nearly three times higher than that of alginate hydrogels. The mechanical properties of the PCH and PANI–PCH films were evaluated using both compressive and tensile stress–strain tests. As shown in the tensile stress–strain data (Figure 4b), the PCH (PVA–H_2_SO_4_ chemical hydrogel) film demonstrated exceptional elasticity, capable of stretching up to 290% under a stress of 0.1 MPa. These films also maintain approximately 90% water content and exhibit an ionic conductivity of 0.082 S cm^−1^, underscoring their potential as flexible electrolyte components. When employed in a symmetric supercapacitor configuration utilizing polyaniline (PANI) electrodes and GA-crosslinked PVA-H_2_SO_4_ electrolyte, the device achieved an impressive areal capacitance of 488 mF cm^−2^, while retaining approximately 90% of its capacitance after 7000 cycles (Figure 4c). To further enhance the mechanical robustness and functionality of hydrogel electrolytes, Huang et al. developed a class of vinyl hybrid silica nanoparticle (VSNP)-crosslinked polyacrylamide (PAAm) hydrogels (Figure 5a) [34]. The incorporation of VSNPs reinforces the polymer network and acts as stress buffers, facilitating the dynamic recombination of polymer chains under mechanical deformation. These VSNP–PAAm hydrogels demonstrate exceptional stretchability, capable of elongating up to 1500% without cracking, and achieve ionic conductivities approaching 17 mS cm^−1^ when swollen with electrolyte (Figure 5b). When utilized as electrolytes in supercapacitors with polypyrrole (PPy) electrodes, these hydrogels exhibit both high stretchability—up to 1000%—and significant compressibility, reaching up to 50% deformation (Figure 5c). Such properties highlight their potential for next-generation flexible supercapacitors capable of enduring extreme mechanical strains.

Additionally, montmorillonite (MMT)/PVA hydrogel electrolytes have enhanced thermal stability and antifreezing capability. The lamellar MMT structure promotes ion conduction and strengthens thermal properties. By adjusting dimethyl sulfoxide content, hydrogen bonding in water is weakened, lowering the freezing point. As a result, supercapacitors using MMT/PVA hydrogel electrolytes maintained stable capacitance across a wide temperature range [48,49].

#### 2.1.3. Flexible Supercapacitors with Hydrogel Electrolytes

Flexible supercapacitors are critical components for powering wearable and portable electronic devices. Among various electrolyte materials, poly(vinyl alcohol) (PVA)-based hydrogels have gained widespread adoption owing to their low cost, non-toxicity, and inherent chemical stability. Nonetheless, their electrochemical performance remains limited by inherent material constraints. PVA hydrogels often suffer from poor water retention and low ionic conductivity, attributable to their long carbon backbone and insufficiently hydrophilic side chains. Although freshly prepared PVA hydrogels mixed with electrolytes such as H_2_SO_4_, H_3_PO_4_, or KOH can attain ionic conductivities in the range of 10^−4^ to 10^−1^ S cm^−1^, rapid water evaporation causes significant conductivity loss within a few days, thereby reducing device stability [50,51,52,53,54,55,56,57,58,59]. Additionally, as the PVA gels solidify, they tend to become rigid, impairing the flexibility and mechanical integrity of energy storage devices. Such issues lead to rapid self-discharge, mechanical degradation, and ultimately limit the long-term stability and applicability of PVA-based supercapacitors [49].

**Figure 6:**
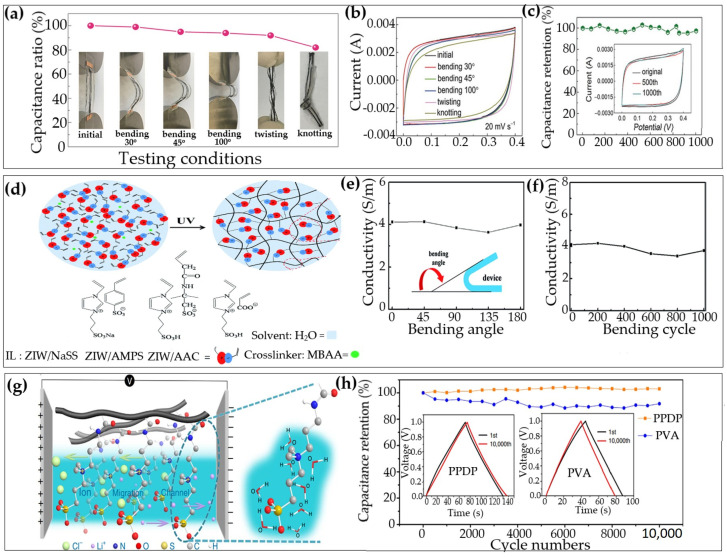
Representative examples of flexible hydrogel electrolytes for fiber-based supercapacitors. (**a**–**c**) Fabrication and structural features of a fiber-shaped supercapacitor utilizing a crosslinked PVA hydrogel electrolyte, reproduced with permission from ref. [61] Wiley & Sons 2017. (**d**) Synthetic pathway of a poly(ionic liquid) (ZIW/AMPS) designed as a flexible electrolyte material. (**e**,**f**) Ionic conductivity of the hydrogel under various bending conditions, reproduced with permission from ref. [46] Royal Society of Chemistry 2015. (**g**) Illustration of a polyzwitterionic hydrogel employed as an electrolyte in supercapacitors. (**h**) Comparative electrochemical performance of the polyzwitterionic hydrogel versus conventional PVA-based electrolytes [62]. Open access.

To overcome these limitations, substantial efforts have focused on chemically and physically modifying PVA hydrogels. One effective approach involves chemical crosslinking of PVA with glutaraldehyde (GA), which significantly enhances elasticity, water retention, and electrochemical performance. For example, Wang et al. developed a GA-crosslinked PVA–H_2_SO_4_ hydrogel film for a symmetric PANI supercapacitor, achieving a water content of approximately 90%, an ionic conductivity of 0.082 S cm^−1^, and an areal capacitance of 488 mF cm^−2^, while maintaining about 90% capacitance retention after 7000 electrochemical cycles [49]. Similarly, Zang et al. fabricated a three-layer composite supercapacitor using polypyrrole (PPy) grown on chemically crosslinked PVA–H_2_SO_4_ hydrogel (CPH). The device demonstrated a tensile strength of 20.83 MPa, a fracture strain of 377%, and delivered a volumetric capacitance of 13.06 F cm^−3^ along with an energy density of 1160.9 μWh cm^−3^. Notably, the device retained 97.9% of its initial capacitance after 10,000 folding and charge–discharge cycles [60]. Extending this concept, Hu et al. designed fiber-shaped supercapacitors utilizing MXene-coated silver-plated nylon fibers coupled with GA-crosslinked PVA–H_2_SO_4_ hydrogel. These fiber-shaped devices exhibited an areal capacitance of 328 mF cm^−2^ and demonstrated excellent mechanical flexibility, maintaining performance under bending, twisting, and knotting conditions (Figure 6a–c) [61]. Beyond PVA, other synthetic hydrogels have been explored. Zhou et al. synthesized a poly(ionic liquid) hydrogel (ZIW/AMPS) with high toughness, flexibility, and self-recovery. It exhibited ionic conductivity over 1 S cm^−1^ at room temperature and low activation energy, ideal for flexible energy storage Figure 6d–f [46]. Peng et al. developed a polyzwitterionic hydrogel electrolyte based on poly(propylsulfonate dimethylammonium propylmethacrylamide) (PPDP) for graphene-based supercapacitors. Its strong water affinity and ion separation capability enabled a capacitance of 300.8 F cm^−3^ at 0.8 A cm^−3^, with only 14.9% degradation at 20 A cm^−3^. The device retained 103% capacitance after 10,000 cycles [62] Figure 6b,g,h. Li et al. reported a PAAm–LiCl hydrogel with ionic conductivities ranging from 3.8 to 8.1 S cm^−1^. Carbon nanotube supercapacitors using this electrolyte achieved 99.3 F cm^−3^ (198.6 mF cm^−2^) and maintained performance after 5000 bending, knotting, and kneading cycles. The device also exhibited an extended self-discharge time of over 10 h [63].

Compared to traditional PVA-based gels, these innovative hydrogel electrolytes provide enhanced ionic conductivity, water retention, and mechanical strength. As the development of flexible supercapacitors advances, solid-state and quasi-solid-state hydrogel electrolytes are becoming increasingly important—acting both as ion transport mediums and mechanical buffers.

#### 2.1.4. Hydrogel Electrolyte-Based Supercapacitors with Additional Functions

**Figure 7:**
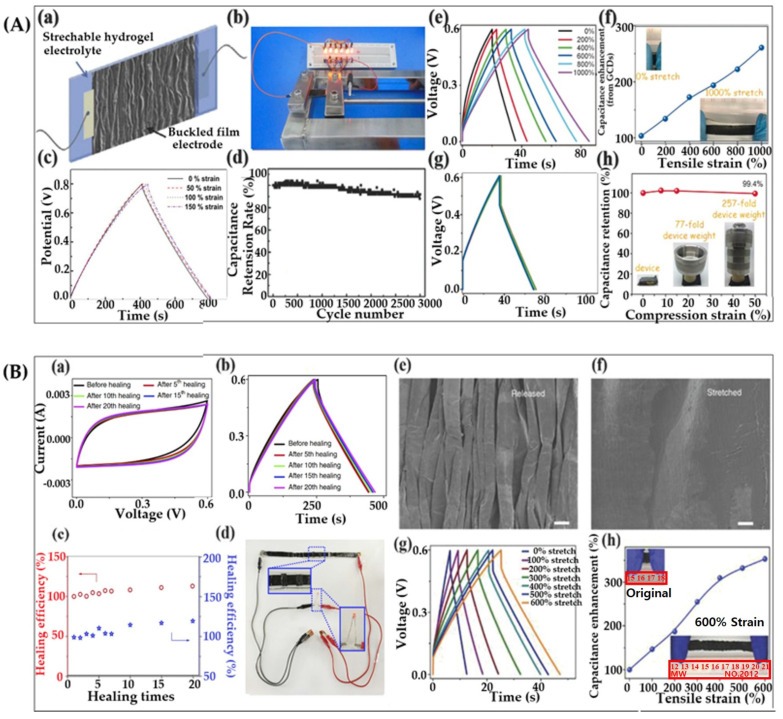
Stretchable, compressible, and self-healing hydrogel electrolytes for flexible supercapacitors. (**A**) Na_2_SO_4_–aPUA/PAAm and PAAm hydrogel electrolytes: (**a**,**b**) Preparation of Na_2_SO_4_–aPUA/PAAm hydrogel; (**c**,**d**) electrochemical performance of supercapacitors using this electrolyte, reproduced with permission from ref. [69] Elsevier 2015. (**e**,**f**) Electrochemical and mechanical stretchability of PAAm-based devices; (**g**,**h**) compressibility and corresponding electrochemical behavior, reproduced with permission from ref. [34] Wiley & Sons 2017. (**B**) Dual-crosslinked PAA-VSNP hydrogel electrolyte for stretchable and self-healing supercapacitors: (**a**–**d**) Self-healing and electrochemical performance; (**e**–**h**) stretchability and device performance [67], Nature 2016 (open access).

As the demand for multifunctional and adaptive electronics grows, supercapacitors are evolving beyond simple energy storage devices. They are now expected to operate reliably under mechanical deformation, environmental stress, and even damage, making multifunctional materials a necessity in next-generation energy systems. Conventional supercapacitors lack the ability to respond to mechanical, chemical, or physical stimuli, limiting their integration into smart, wearable, and reconfigurable electronics [64,65,66,67,68,69]. Traditional electrolytes are not designed to accommodate stretchability, compressibility, or self-repair, which are essential for devices operating in dynamic environments. Hydrogel electrolytes, owing to their tunable chemistry and inherent mechanical flexibility, have emerged as promising platforms for the integration of multifunctional features into supercapacitors. Their soft, hydrated polymer networks can be engineered to exhibit properties such as stretchability, compressibility, and self-healing—attributes that are essential for wearable and deformable energy storage devices (Figure 7A) [34,69]. A notable approach involves the development of stretchable hydrogel electrolytes. For instance, Tang et al. fabricated a supercapacitor utilizing a Na_2_SO_4_–aPUA/PAAm hydrogel with intrinsic stretchability exceeding 1000%. Their innovative “prestrain–stick–release” technique allowed for strong adhesion and maintained an ionic conductivity of 0.036 S cm^−1^. The device demonstrated a specific capacitance of 478.6 mF cm^−2^ at 0.5 mA cm^−2^ and retained 91.5% of its capacitance after 3000 cycles under 150% strain, highlighting its mechanical robustness and electrochemical stability [69]. Similarly, Huang et al. reinforced PAAm hydrogels with vinyl hybrid silica nanoparticles (VSNPs), forming a dual-network structure stabilized by covalent and hydrogen bonds. This VSNP–PAAm hydrogel displayed super-stretchability (>1000%), high compressibility (50%), and a 2.6-fold increase in capacitance with an impressive retention rate of 99.4% [34]. Interestingly, the underlying mechanisms responsible for stretchability and self-healing largely share commonalities, relying on the synergy between permanent covalent crosslinking and reversible interactions such as hydrogen bonding and ionic interactions [67,68]. For example, Huang et al. designed a PAA-based hydrogel crosslinked through both VSNPs and hydrogen bonds, achieving remarkable stretchability exceeding 3700% and highly robust self-healing capabilities (Figure 7B) [67].

**Figure 8:**
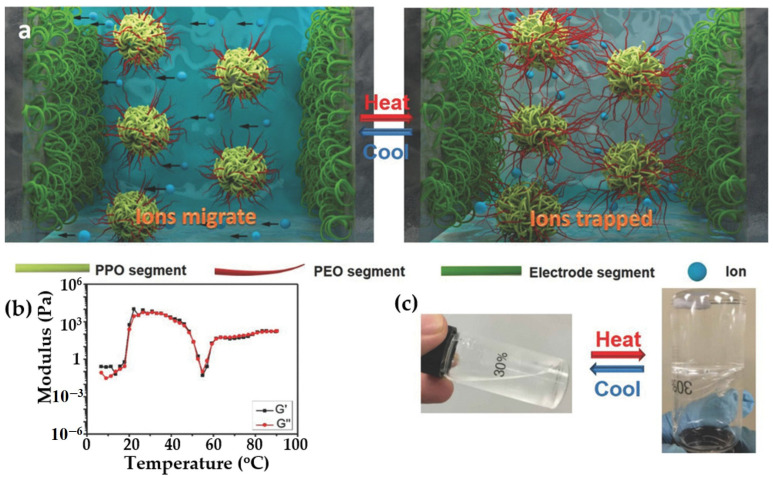
Thermoresponsive gel electrolyte for flexible supercapacitors. (**a**) Schematic of a thermoresponsive gel system based on Pluronic used as an electrolyte. (**b**) Temperature-dependent rheological behavior of a 30 wt% Pluronic solution, showing storage (G′) and loss (G″) moduli. (**c**) Photographs of the Pluronic solution in sol and gel states, reproduced with permission from ref. [70], Wiley & Sons 2016.

Conventional hydrogel electrolytes often exhibit limited responsiveness to environmental stimuli such as temperature and generally do not participate actively in redox reactions. This restricts their utility in the development of smart energy storage systems that demand functionalities like self-regulation, biodegradability, or integrated energy conversion capabilities. To address these limitations, research has increasingly focused on the development of advanced hydrogel systems, including thermoresponsive hydrogels, biodegradable materials, and redox-active electrolytes. These innovations enable hydrogels to adapt dynamically to ambient conditions, safely degrade in biological environments, or actively participate in electrochemical processes—thus significantly expanding the functional scope of supercapacitors. For instance, Shi et al. engineered a thermoresponsive hydrogel composed of Pluronic copolymer (PEO–PPO–PEO), which undergoes reversible phase transitions between sol and gel states depending on temperature (Figure 8) [70].

Below 20 °C, the hydrogel remains in a sol state, allowing ion transport; above 80 °C, it gels and restricts ion mobility. This behavior enables self-protection: the supercapacitor performs normally at room temperature (~100 F g^−1^), but its capacitance drops to ~10 F g^−1^ at high temperatures, effectively halting operation. Biodegradable hydrogel electrolytes also show promise. Moon et al. reported a NaCl–agarose hydrogel crosslinked via hydrogen bonding, forming a porous 3D scaffold with high water content and ion mobility [71]. A MnO_2_-based supercapacitor using this hydrogel achieved 286.9 F g^−1^ and retained over 80% capacitance at 100 mV s^−1^. The device maintained performance under bending (radius: 1.16 cm), demonstrating mechanical flexibility and biodegradability. Lee et al. extended this concept to transient electronics, using agarose hydrogel electrolytes in micro-supercapacitors with water-soluble metal electrodes (e.g., Mo, Fe, W) and PLGA substrates [72]. These devices delivered 1.6 mF cm^−2^ areal capacitance and biodegraded in phosphate-buffered saline over days to weeks, depending on conditions—highlighting their potential for resorbable biomedical applications.

Redox-active hydrogel electrolytes offer an additional functional benefit. Tang et al. prepared PVA-based hydrogels containing the ionic liquid [BMIM]Cl, lactic acid, and LiBr to enable Br^−^/Br_3_^−^ redox reactions [73]. They used acidic hydrogels as catholytes and neutral hydrogels as anolytes to suppress side reactions and broaden the operating voltage window. The resulting supercapacitor reached 1.6 V, with an energy density of 16.3 Wh kg^−1^ and a power density of 932.6 W kg^−1^ (measured at 2 A g^−1^). It retained 93.4% of its capacitance after 10,000 cycles and maintained performance when bent from 0° to 180°.

Table 1 and Table 2 and present the typical performance metrics and additional functionalities of the supercapacitors, respectively.

## 3. Applications of Hydrogel-Derived Materials for Energy Conversion Devices

**Figure 9:**
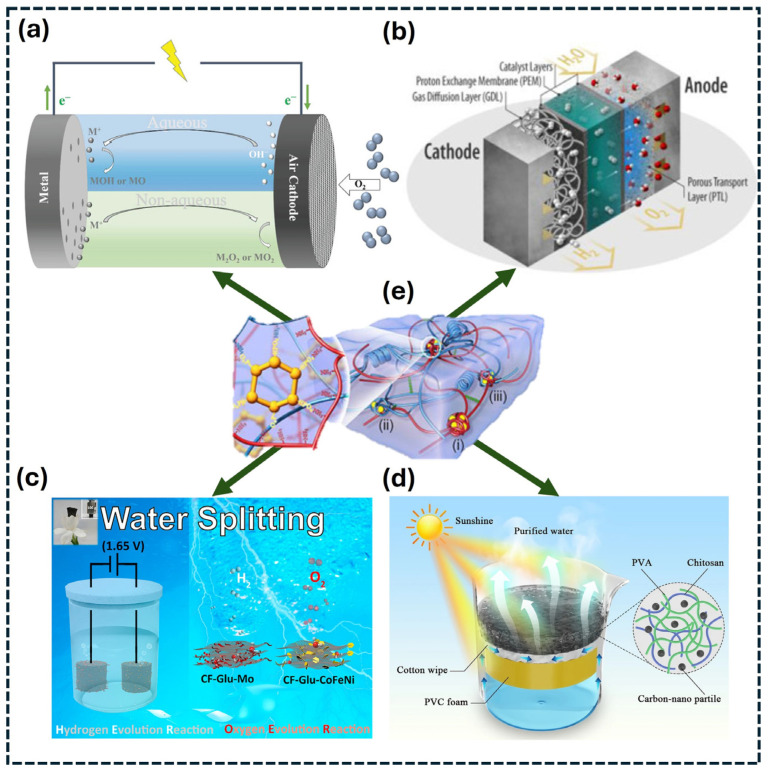
Schematic overview of hydrogel-based materials utilized in energy conversion devices. Adapted and redrawn from references [87,88,89,90,91]. (**a**) Schematic representation of metal–air batteries with aqueous (upper, blue) and non-aqueous (below, green) electrolytes [87]. Open access. (**b**) Fuel cells, reproduced with permission from ref. [88], Elsevier 2022. (**c**) Water splitting, reproduced with permission from ref. [89]. Elsevier 2021. (**d**) Schematic illustration of regular double-layer evaporation system [90], open access. (**e**) Hydrogels, reproduced with permission from ref. [91], Elsevier 2020.

Energy conversion devices—including fuel cells, metal–air batteries, solar cells, and water-splitting electrolyzers—are fundamental components of sustainable energy systems (Figure 9). These technologies primarily depend on key electrocatalytic processes such as the oxygen reduction reaction (ORR), oxygen evolution reaction (OER), and hydrogen evolution reaction (HER). These electrochemical reactions are critical for facilitating efficient energy conversion between chemical and electrical forms, underpinning the development of clean and renewable energy solutions.

Recent advances have highlighted the versatility of hydrogel-derived materials in energy conversion applications. Hydrogels can serve as electrocatalyst precursors by undergoing pyrolysis to form porous carbon frameworks embedded with metal or metal oxide nanoparticles, offering high surface area, electrical conductivity, and well-dispersed active sites. Additionally, they can be engineered into flexible, ion-conductive membranes for use as electrolytes in fuel cells and other electrochemical systems. Furthermore, the integration of hydrogels with functional nanomaterials—such as graphene or metal-organic complexes—enables the development of hybrid architectures that combine superior catalytic performance with enhanced mechanical integrity. These developments position hydrogels as a versatile platform for advancing the performance and integration of energy conversion technologies.

### 3.1. Metal–Air Batteries

**Figure 10:**
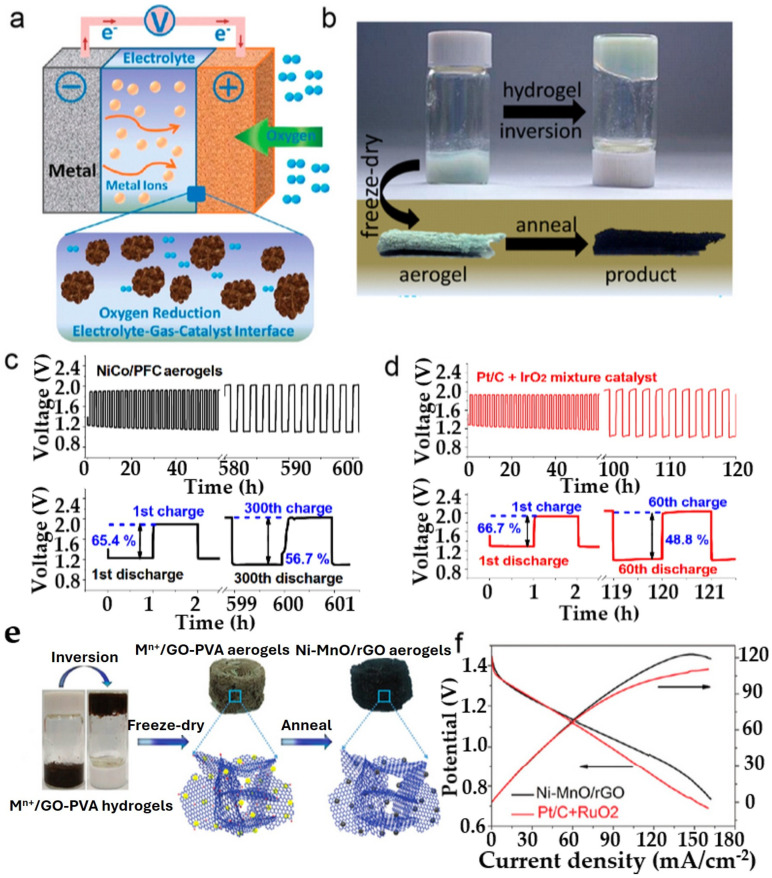
Hydrogel-derived materials for Zn–air batteries. (**a**) Schematic of the working principle of a metal–air battery, highlighting the liquid–gas–solid interface in the air electrode, reproduced with permission from ref. [92], Royal Society of Chemistry 2012. (**b**) Fabrication of 3D NiCo/PFC hydrogel [95] (**c**,**d**) charge/discharge profiles of Zn–air batteries using NiCo/PFC aerogels and conventional Pt/C + IrO_2_ catalysts, respectively, reproduced with permission from ref. [95], American Chemical Society 2016. (**e**) Preparation of Ni–MnO/rGO aerogels; (**f**) discharge polarization and power density curves of Zn–air batteries with Ni–MnO/rGO and commercial Pt/C + RuO_2_ air electrodes, redrawn and reproduced with permission from ref. [96], Wiley & Sons 2018.

Metal–air batteries, particularly zinc–air (Zn–air) systems, have attracted significant attention as promising candidates for next-generation energy storage solutions due to their extraordinarily high theoretical energy densities, reaching approximately 1350 Wh kg^−1^ (excluding oxygen consumption) [92,93] (Figure 10a). Despite their prospective advantages, practical deployment remains challenging, primarily due to sluggish kinetics at the air cathode and inefficient utilization of the zinc anode. The air electrode, which facilitates the oxygen reduction reaction (ORR) and oxygen evolution reaction (OER), often suffers from high overpotentials, resulting in limited energy efficiencies typically ranging between 55% and 65% [94]. Hydrogels, distinguished by their three-dimensional interconnected porous structures, large surface areas, and tunable chemical compositions, have emerged as versatile precursors for fabricating advanced electrocatalyst frameworks and functional membranes. Their macromolecular networks are capable of effectively trapping electrolytes and can be processed into membranes with excellent ionic conductivity and ion-exchange capabilities—attributes that prove advantageous for constructing both active electrodes and electrolyte components in energy conversion and storage devices [92].

Innovative strategies have leveraged hydrogel systems to develop bifunctional catalysts with enhanced activity and stability. For instance, Fu et al. employed K_2_Ni(CN)_4_/K_3_Co(CN)_6_–chitosan (CS) hybrid hydrogels to derive catalysts with superior bifunctional activity for Zn–air batteries (Figure 10b) [95]. The hydrogel matrices were stabilized via coordination bonds between metal ions and amino groups on chitosan, as well as hydrogen bonding between hydroxyl groups and cyanometallates. Upon pyrolysis, uniform NiCo nanoparticles were embedded within a porous carbon framework, resulting in catalysts exhibiting comparable ORR and OER activities to commercial Pt/C and IrO_2_ catalysts. Notably, Zn–air batteries employing these catalysts demonstrated remarkable durability, operating for approximately 600 h at a current density of 10 mA cm^−2^ with minimal voltage loss, outperforming traditional Pt/C + IrO_2_ systems, which experienced a voltage drop of approximately 0.5 V after only 120 h (Figure 10c,d) [95]. In parallel, Fu et al. designed a Ni–MnO/rGO bifunctional catalyst derived from a 3D porous hydrogel composed of GO–PVA hybrid hydrogel precursors loaded with manganese and nickel salts (Figure 10e) [96]. This hydrogel was formed via a sol–gel polymerization process involving PVA, graphene oxide, manganese acetate, and nickel acetate, stabilized by hydrogen bonding and metal–GO complexation. Subsequent pyrolysis yielded Ni and MnO nanoparticles uniformly anchored on reduced graphene oxide (rGO) nanosheets. The resulting Ni–MnO/rGO catalyst exhibited excellent ORR and OER activity, closely matching the performance of benchmark catalysts such as Pt/C and RuO_2_. Zn–air batteries utilizing this catalyst achieved stable charge/discharge cycling over 100 cycles, surpassing the performance of comparable systems based on Pt/C + RuO_2_, and demonstrating superior long-term stability (Figure 10f) [96].

### 3.2. Fuel Cells

**Figure 11:**
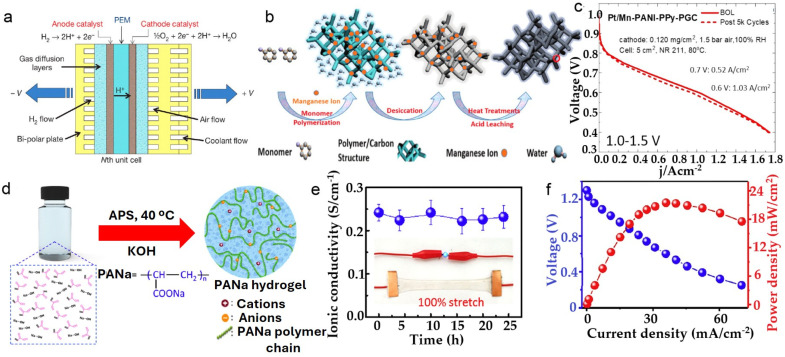
Hydrogel-based components for fuel cell applications. (**a**) Schematic of fuel cell operation and the liquid–gas–solid interface in the air electrode, reproduced with permission from ref. [97], Nature 2012. (**b**) Synthesis of polymer hydrogel-derived porous graphitic carbon supporting Pt particles. (**c**) Discharge curves of fuel cells using Pt/Mn-PANI-PPy-PGC air electrodes before and after 5000 cycles, reproduced with permission from ref. [98], Royal Society of Chemistry 2019. (**d**) Preparation of PANa alkaline hydrogel membrane. (**e**) Ionic conductivity of PANa hydrogel under static and stretched conditions, with inset showing its use in powering an LED circuit. (**f**) Polarization and power density curves of a direct ethanol fuel cell (DEFC) using ethanol and air as fuels, reproduced with permission from ref. [99], Elsevier 2019.

Fuel cells are electrochemical devices that convert the chemical energy of fuels—such as hydrogen, methanol, or ethanol—into electricity through redox reactions. In a typical fuel cell, hydrogen oxidation occurs at the anode, while the oxygen reduction reaction (ORR) takes place at the cathode. These reactions are separated by a proton or cation exchange membrane (Figure 11a) [97] The core of the fuel cell is the membrane electrode assembly (MEA), which is compressed between bipolar plates that facilitate the transport of reactants and coolants while collecting the generated current.

Fuel cells are an efficient and clean way to produce energy, but their commercial use faces major obstacles. The main problems are the high cost and limited durability of platinum (Pt) catalysts and the poor stability of common carbon supports. Pt is the best catalyst for both the oxygen reduction reaction (ORR) and hydrogen oxidation, yet it is expensive and prone to degradation, especially in acidic, hot, or very humid conditions. Common carbon supports like XC-72 and Ketjen black are mostly amorphous and poorly graphitized, making them vulnerable to corrosion and structural breakdown during long-term operation. Together, these issues reduce catalyst performance and shorten fuel cell lifetime [100]. Hydrogels present a promising solution to these limitations due to their tunable molecular structure and capacity to serve as precursors for three-dimensional porous graphitic carbon frameworks. These hydrogel-derived carbons exhibit high surface areas, uniform heteroatom doping, and improved electrical conductivity, making them ideal supports for dispersing Pt nanoparticles or developing non-precious metal catalysts. Moreover, hydrogel-based membranes possess excellent water retention, ionic conductivity, and chemical stability, enabling operation in both acidic and alkaline environments—thus offering a viable alternative to commercial membranes such as Nafion, which is costly and prone to dehydration and crossover issues in direct alcohol fuel cells. To address catalyst degradation and enhance electrochemical stability, Qiao et al. developed a hybrid hydrogel-derived carbon support composed of polyaniline (PANI) and polypyrrole (PPy) for Pt nanoparticles (Figure 11b) [98]. The nitrogen-rich aromatic structures in PANI were transformed into nitrogen-doped graphitic carbon upon pyrolysis, while PPy contributed to the formation of highly folded and contorted graphitic domains. The inclusion of manganese (Mn) precursors during polymerization further improved graphitization and corrosion resistance. The resulting Pt-loaded catalyst demonstrated exceptional electrochemical stability, with negligible voltage decay after 5000 cycles at a current density of 1.5 A cm^−2^—surpassing the U.S. Department of Energy durability target of less than 30 mV voltage loss (Figure 11c). In efforts to reduce reliance on precious metals, non-precious metal catalysts (NPMCs) have been explored, typically synthesized via pyrolysis of heteroatom-doped polymers such as polyaniline containing Co or Fe. These catalysts develop active M–N–C (metal–nitrogen–carbon) sites, which catalyze ORR effectively. PANI-derived Fe/Co catalysts could achieve stable operation in fuel cells for over 700 h at 0.4 V, showcasing their potential as cost-effective alternatives [101]. Despite these advances, hydrogel-derived non-precious catalysts remain underexplored, and future research should focus on optimizing nitrogen content, metal precursor distribution, and pyrolysis parameters to further enhance ORR activity and durability.

Regarding membrane technology, while Nafion remains the industry standard due to its high ionic conductivity, it is expensive, prone to dehydration, and susceptible to crossover of methanol or ethanol—conditions that can poison catalysts in direct alcohol fuel cells. To overcome these limitations, Wang et al. introduced a flexible direct ethanol fuel cell utilizing a sodium polyacrylate (PANa) hydrogel as the alkaline membrane, paired with a porous N,S-co-doped carbon catalyst as the cathode (Figure 11d) [99]. The superabsorbent PANa membrane maintained high ionic conductivity even under mechanical deformation (Figure 11e), enabling the device to sustain performance during 180° bending tests (Figure 11f). The system achieved a power density of 21.48 mW cm^−2^ and an energy density of 1.41 mWh cm^−2^, surpassing performance metrics of many conventional rigid fuel cells and batteries. This work illustrates the potential of hydrogel-based membranes to enable flexible, high-performance energy conversion devices suitable for wearable and portable applications.

### 3.3. Water-Splitting Electrolyzers

**Figure 12:**
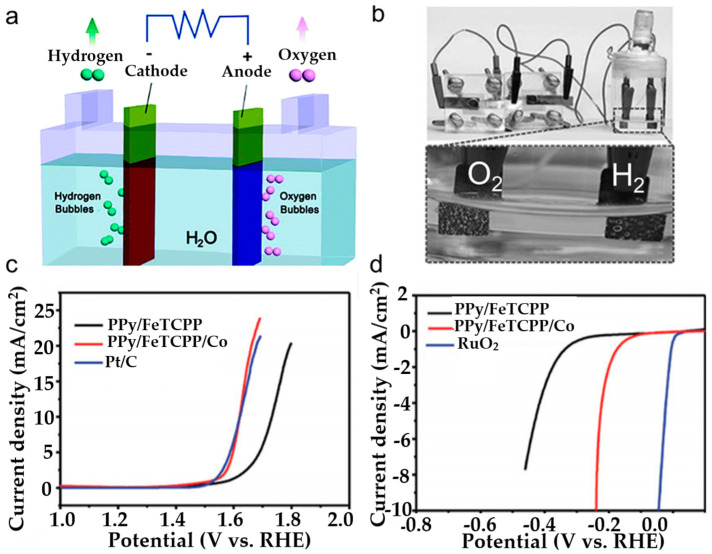
Hydrogel-based systems for water electrolysis. (**a**) Schematic of a water electrolyzer for overall water splitting, reproduced with permission from ref. [102], Royal Society of Chemistry 2015. (**b**) Photographs of the device setup. (**c**,**d**) Linear sweep voltammetry (LSV) curves for oxygen evolution reaction (OER) and hydrogen evolution reaction (HER) using PPy/FeTCPP, PPy/FeTCPP/Co, and RuO_2_ catalysts in 0.1 M KOH, redrawn and reproduced with permission from ref. [104], Wiley & Sons 2017.

Electrochemical water splitting stands as a promising and scalable approach for sustainable hydrogen production, a clean energy vector with broad potential applications. The process involves two distinct half-reactions: the hydrogen evolution reaction (HER) occurring at the cathode and the oxygen evolution reaction (OER) at the anode (Figure 12a) [102]. Both reactions are proton-coupled electron transfer processes that are integral to various technologies, including hydrogen fuel cells and renewable energy storage systems. Despite its advantages, the widespread adoption of large-scale water splitting faces significant hurdles, primarily due to the lack of efficient, cost-effective, and durable electrocatalysts. Noble metals such as platinum (Pt) and iridium/ruthenium (Ir/Ru) are currently the most effective catalysts for HER and OER, respectively; however, their high cost and limited availability substantially restrict their practical commercial deployment [103]. Moreover, bifunctional catalysts capable of efficiently catalyzing both HER and OER simultaneously are rare, complicating system integration and increasing overall costs.

Hydrogels have emerged as versatile precursors for making catalysts because their three-dimensional networks let researchers control porosity, surface area, and dopant distribution—key factors for catalytic activity. After pyrolysis, hydrogel-derived materials can form conductive carbon frameworks doped with transition metals, yielding bifunctional catalysts for overall water splitting. Hydrogels can also be made into flexible, ion-conductive membranes, which broadens their use in water electrolysis systems. Recent work shows clear progress: for example, PPy/FeTCPP/Co hydrogels were formed by supramolecular cross-linking with FeTCPP acting as both dopant and cross-linker, then pyrolyzed to produce porous carbon frameworks coated onto carbon paper electrodes for water electrolysis (Figure 12b). This strategy demonstrates the potential of hydrogel-derived materials to produce efficient, low-cost, and flexible catalyst electrodes for large-scale hydrogen production.

The device showed simultaneous H_2_ and O_2_ generation with a stoichiometric 2:1 ratio, confirmed by gas chromatography. It maintained catalytic durability for over 12 h and exhibited overpotentials of 240 mV for HER and 380 mV for OER (Figure 12c,d). Moreover, the PPy/FeTCPP/Co-derived catalyst also demonstrated good oxygen reduction reaction (ORR) activity, enabling its use in a self-powered water-splitting system driven by a zinc–air battery. This multifunctionality—HER, OER, and ORR—highlights the potential of hydrogel-derived materials to serve as trifunctional electrocatalysts [105].

### 3.4. Hydrogels in Solar Cells

**Figure 13:**
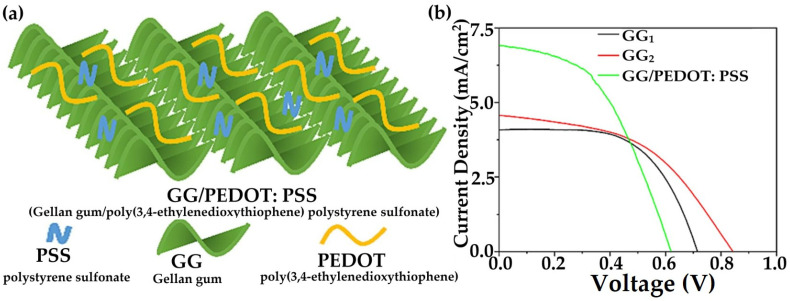
(**a**) Structural model of the GG/PEDOT: PSS gel electrolyte. (**b**) Current–voltage (I–V) characteristics of dye-sensitized solar cells (DSSCs) using different gel electrolytes, redrawn and reproduced with permission from ref. [109], Elsevier 2024.

Solar energy remains one of the most abundant and sustainable sources of renewable power. Among various photovoltaic technologies, dye-sensitized solar cells (DSSCs) have garnered considerable interest owing to their low cost, facile fabrication processes, and the potential for flexible, transparent, and lightweight applications. The efficiency and long-term stability of DSSCs are heavily influenced by the properties of their electrolyte systems, which play a central role in facilitating charge transport between the photoanode and the counter electrode. Traditional DSSCs typically utilize organic solvent-based liquid electrolytes, which, despite their high ionic conductivity and efficiency, are plagued by significant limitations. These include high volatility, flammability, and the risk of leakage, which compromise device safety and operational stability. Furthermore, these solvents can induce corrosion of electrodes and conductive substrates, leading to performance deterioration over time [106,107,108]. Water-based electrolytes offer a safer alternative, but their application is hampered by inherently lower open-circuit voltages (usually in the range of 0.4–0.6 V), thus constraining the overall power conversion efficiency of the device. Hydrogels, with their quasi-solid-state nature, present a promising solution to these challenges by combining the ionic conduction properties of liquid electrolytes with the mechanical robustness of solids. Their three-dimensional polymeric networks are capable of retaining substantial amounts of water and electrolyte ions, effectively reducing evaporation and leakage while maintaining sufficient ion mobility. This unique combination makes hydrogels highly suitable as electrolyte materials for DSSCs, potentially enhancing both device performance and operational durability. Recent advancements have demonstrated the successful integration of hydrogel-based electrolytes in DSSCs, contributing to improvements in efficiency, stability, and environmental sustainability. For example, Zarate et al. employed a zinc–galactomannan hydrogel as a green, eco-friendly electrolyte, achieving an open-circuit voltage of 750 mV. This evidence suggests that hydrogel systems can rival conventional electrolytes while offering substantial ecological and safety benefits. Similarly, Unlu et al. developed a gellan gum/poly(3,4-ethylenedioxythiophene):polystyrene sulfonate (GG/PEDOT:PSS) hydrogel, immersed within an I^−^/I_3_^−^ redox couple and utilized as a quasi-solid electrolyte. Among three configurations—GG1 (2 mm), GG2 (1 mm), and GG/PEDOT:PSS—the hydrogel incorporating PEDOT:PSS (Figure 13) demonstrated the highest short-circuit current density (Jsc) of 6.92 mA cm^−2^, outperforming the other configurations [109]. This enhancement was attributed to the conjugated polymeric 3D gel network, which facilitated efficient electron transfer and optimized ion mobility, thereby underscoring the promising role of hydrogel-based electrolytes in advancing DSSC technology.

These findings underscore the potential of hydrogel-based electrolytes to enhance the performance, safety, and environmental sustainability of DSSCs. As summarized in Table 3 of the original study, hydrogel-integrated solar cells have achieved power conversion efficiencies of up to 14%, making them competitive with more conventional photovoltaic technologies.

Hydrogel-derived materials have demonstrated remarkable versatility and functionality in a wide range of energy conversion devices, including metal–air batteries, fuel cells, water-splitting electrolyzers, and solar cells. Their unique properties—such as high water retention, tunable porosity, ionic conductivity, and structural flexibility—enable efficient charge transport, enhanced catalytic activity, and improved device stability. These characteristics make hydrogels promising candidates for next-generation sustainable energy technologies.

To provide a comprehensive overview, Table 4 summarizes various hydrogel-based materials and their corresponding applications in energy conversion devices, highlighting their key roles, advantages, and performance characteristics.

However, challenges remain in terms of long-term durability, large-scale fabrication, and integration with existing device architectures. Future research should focus on optimizing the physicochemical properties of hydrogels, exploring hybrid composites, and developing cost-effective synthesis strategies to fully realize their potential in practical applications.

## 4. Challenges and Future Perspectives

Hydrogels have attracted considerable interest for various applications, such as energy storage, sensors, solar cells, supercapacitors, and energy conversion systems, due to their distinctive properties. Nevertheless, several critical limitations hinder their wider adoption in energy-related devices.

1. Strength and Durability: While hydrogels are known for their exceptional flexibility, they often lack sufficient mechanical strength and durability, especially under extreme conditions like high temperatures, humidity, or mechanical stress. This leads to performance decline, restricting their application in long-term uses such as wearable electronics and energy storage systems. To overcome these limitations, researchers are developing hybrid hydrogels that incorporate stronger materials, such as polymers or metallic nanoparticles, to enhance their mechanical properties. The integration of highly conductive materials like carbon nanotubes and graphene into hydrogel matrices has demonstrated potential for improving both mechanical strength and electrical conductivity. In addition, self-healing hydrogels, capable of autonomously repairing damage, present a promising solution to the durability issues associated with hydrogels. By incorporating dynamic covalent bonds or reversible cross-linking mechanisms, these hydrogels can restore their functionality after exposure to mechanical or environmental stress, which is essential for ensuring the long-term reliability of devices. These modifications aim to improve durability while maintaining flexibility, making hydrogels more suitable for demanding applications.
2. Biodegradability and Environmental Concerns: Hydrogels are generally regarded as environmentally friendly, but the byproducts of certain synthetic hydrogels may pose long-term ecological risks. Ongoing research aims to create fully biodegradable hydrogels that can degrade safely in natural settings. These biodegradable hydrogels could minimize the environmental impact of synthetic hydrogels while maintaining their beneficial properties. Recently, biodegradable and biobased hydrogels, such as those derived from polysaccharides, have gained a lot of attention. Researchers are exploring natural polymers like cellulose and alginate to develop hydrogels that break down efficiently without releasing harmful residues.
3. Ionic Conductivity: The ionic conductivity of hydrogels is generally lower than that of conventional solid-state electrolytes or metal-based conductors, despite being suitable for specific applications like supercapacitors. A major challenge is enhancing the ion transport within the hydrogel matrix while maintaining other critical properties such as biocompatibility and environmental sustainability. To address this challenge, researchers are exploring innovative strategies such as incorporating conductive nanoparticles, optimizing hydrogel composition, or designing hierarchical structures to enhance ion mobility. Balancing these improvements with sustainability and biocompatibility remains a critical focus for advancing hydrogel-based technologies.
4. Manufacturing and Scalability: Scalability remains a major challenge for hydrogel-based devices, particularly in large-scale energy storage applications. Producing these systems on a large scale while ensuring uniformity and consistent performance is often difficult. Advancements in manufacturing techniques, such as 3D printing and automated assembly lines, could help improve scalability. Advances in 3D printing and other advanced fabrication techniques are facilitating precise control over hydrogel structures, enabling the development of hydrogels with customized properties for specific energy applications. This includes enhancing pore structures to improve ion transport and modifying polymer networks to enhance mechanical strength. Additionally, optimizing the hydrogel formulation to enhance its mechanical properties and stability can lead to more reliable large-scale production. Collaborations with materials scientists and engineers can also drive innovation in this sector to address these challenges effectively. Collaboration among experts in nanotechnology, organic electronics, and biomaterials is essential for advancing next-generation hydrogels for energy storage and conversion systems.

## 5. Conclusions

### 5.1. Summary of Key Points

Hydrogels are a versatile and promising category of materials for energy storage and conversion applications. Their unique properties, including high water content, ionic conductivity, and mechanical flexibility, make them well suited for use in supercapacitors, fuel cells, and solar cells. While challenges such as mechanical strength, scalability, and environmental impact remain, research into composite hydrogels, advanced fabrication methods, and self-healing systems is driving progress toward more durable and efficient devices. The future of hydrogel-based energy technologies depends on interdisciplinary collaboration and innovation in material design, with these materials expected to play a pivotal role in developing sustainable, high-performance next-generation energy systems.

### 5.2. Impact of Hydrogels on the Future of Energy Materials and Devices

The incorporation of hydrogels into energy devices holds the potential to transform the energy industry. When optimized for high-performance applications, hydrogels could enable the creation of sustainable, flexible, and eco-friendly energy storage solutions. Their unique characteristics, such as high water content, ionic conductivity, and biocompatibility, position them as ideal materials for next-generation energy devices, including wearable electronics, flexible sensors, and efficient energy storage systems.

### 5.3. Final Thoughts

The field of hydrogel-based energy materials is advancing rapidly, with a promising future ahead. Innovations in material design, fabrication methods, and functionalization approaches are anticipated to address current limitations, opening up new applications across various domains, from wearable devices to large-scale energy storage systems. By tackling challenges and expanding the scope of hydrogel research, these materials are poised to play a vital role in the creation of next-generation energy systems that are both efficient and sustainable.

## Figures and Tables

**Table 1:** Electrochemical characteristics of EDLC supercapacitors utilizing gel polymer electrolytes (GPEs).

No.	Electrode Material	Gel Polymer Electrolyte (GPE)	Ionic Conductivity	Capacitance (Specific/Areal)	Cycle Stability (Retention)	Ref.
1	PPy@CNT paper	Al-alginate/PAAm hydrogel	29 mS/cm	94.7 mF/cm^2^ @ 0.1 mA/cm^2^	~90% after 3000 cycles	[74]
2	Polypyrrole (PPy)	Agar/HPAAm double network	2 S/m	138.3 mF/cm^2^ @ 0.2 mA	—	[75]
3	Activated carbon	Hydrophobic GPE	31.1 mS/cm	130.3 F/g @ 0.5 A/g	~97% after 5000 cycles @ 1 A/cm^2^	[76]
4	Activated carbon	NaAc-infused crystal-type AAm gel	0.85 S/m	129.7 F/g @ 1.0 A/g	86.6% after 100 cycles	[77]
5	Activated carbon	AG/AAm/LiCl GPE	13 ± 0.8 mS/cm	124.7 F/g @ 0.125 A/g	~80% after 6000 cycles	[78]
6	Polypyrrole (PPy)	PVA/dilute H_2_SO_4_	0.05 S/cm	58.8 F/g	97% after 10,000 cycles @ 1 A/cm^2^	[79]
7	CNT/PPy composite	PAA-based GPE	—	0.22 mF/cm^2^ @ 5 mV/s	—	[80]
8	Activated carbon	Carboxylated chitosan	8.69 × 10^−2^ S/cm	45.9 F/g @ 0.5 A/g	—	[81]
9	Lignin/PAN electrospun nanofibers	Crosslinked lignin GPE	10.35 mS/cm	129.3 F/g @ 0.5 A/g	95% after 10,000 cycles	[82]
10	CNT film	Poly(AMPS-co-DMAAm)/LAPONITE^®^/GO nanocomposite	6.2 mS/cm	180 mF/cm^2^ @ 0.5 mA	91.88% after 10,000 cycles	[83]
11	Activated carbon	PAA-DAC GPE	~8.2 mS/cm	297 mF/cm^2^ @ 0.8 mA	70% after 7000 cycles	[84]
12	PAD/H_2_SO_4_-PANI hydrogel	PAD/H_2_SO_4_ GPE	57 mS/cm	430 mF/cm^2^ @ 0.5 mA	—	[85]
13	CNT paper	PVA/H_2_O/EG/LiCl	2.38 mS/cm @ −40 °C	[—]	88.3% retention after 5000 cycles @ −20 °C	[86]

**Table 2:** Mechanical properties of EDLC supercapacitors incorporating gel polymer electrolytes (GPEs).

No.	Electrode Material	Gel Polymer Electrolyte System	Tensile Strength	Elastic Modulus	Maximum Strain	Capacitance Retention Under Deformation	Ref.
1	PPy@CNT paper	Alginate/PAAm hydrogel	—	429.8 kPa	—	100% retention under 180° bending	[74]
2	Polypyrrole (PPy)	Agar/HPAm double-network	—	—	100–3400%	Not reported	[75]
3	Activated carbon	Hydrophobic GPE	431.9 kPa	—	1150.2%	~100% retention under 90° bending	[76]
4	Activated carbon	NaAc-infused acrylamide gel	—	38.39 MPa	—	105% retention at 350% strain	[77]
5	PPy	PVA/dilute H_2_SO_4_	56 kPa	—	70–110% strain	96% @ 70% strain, 90% @ 110% strain, 73% @ 180° twist	[79]
6	CNT/PPy composite	PAA-based GPE	~16 kPa	—	900%	~100% @ 60% strain, ~10% @ 160° bending	[80]
7	Lignin/PAN nanofibers	Crosslinked lignin GPE	—	—	—	90% @ 60° bending; >98% after repeated stretching (100–300%)	[82]
8	CNT film	Poly(AMPS-co-DMAAm)/LAPONITE^®^/GO nanocomposite	34 kPa	—	1173%	Not reported	[83]
9	PAD/H_2_SO_4_–PANI hydrogel	PAD/H_2_SO_4_ GPE	1.8 MPa	—	2900%	Not reported	[85]
10	CNT paper	PVA/H_2_O/EG/LiCl blend	—	—	300%	81.1% retention after 1000 cycles under 180° bending	[86]

**Table 3:** Summary of hydrogels applications in various types of solar cells.

S.N.	Type of Gel	Characteristics	Type of Solar Cell	Photoelectric Conversion Efficiency	Ref.
01	PANI Hybrid Hydrogel	Nanostructured, flexible, quasi-solid, UV-assisted synthesis, conductivity 0.003–0.02 S/cm	Dye-Sensitized Solar Cell (DSSC) using natural dye (Myrciaria cauliflora)	>2.0%, better than similar systems with the same dye	[110]
02	Graphene Oxide Hydrogel	Quasi-solid, GO forms conductive networks in gelatin, enhancing charge transport	DSSC with NiO@TiO_2_ photoanode	Up to 4.02% (with 5% NiO and 0.1% GO); 1.31% with TiO_2_ only	[111]
03	PAM–MBA Hydrogel	Chemically crosslinked, quasi-solid polysulfide electrolyte, 3D porous network, ionic conductivity 0.093 S/cm	CdS/CdSe QDSC	Up to 4.0%, improved stability and ion transport	[112]
04	PVA/MWCNT/PANI Hydrogel	In situ polymerization, enhanced charge transport via MWCNT network.	DSSC with I^−^/I_3_^−^ gel electrolyte	Up to 2.18% (at 0.75% MWCNT content)	[113]
05	Dextran Hydrogel	Highly conductive, 15 wt% gelator, good pore penetration, fast electron transport, quasi-solid-state	CdS/CdSe QDSC	3.23% (1 sun), up to 4.58% (0.12 sun)	[114]
06	12-Hydroxystearic Acid Hydrogel	Low molecular mass organogelator, gel-liquid transition at 96 °C, enhanced stability, accelerated recombination	CdS/CdSe QDSC	2.40% (1 sun), improved stability vs. liquid electrolyte	[115]
07	Graphene Hydrogel (GH) & GH–CuS Composite	3D porous structure, high conductivity, enhanced catalytic activity, improved stability, low resistance	CdSeTe QDSC	9.85% (GH), up to 10.71% (GH–CuS)	[116]
08	CNT–GH–CuS Composite Hydrogel	Ternary composite, high conductivity, low series resistance (~0.79 Ω), strong catalytic activity	Zn–Cu–In–Se QDSC	14.02% (record efficiency)	[117]
09	Chlorophyll-a/PAM Hydrogel	Bio-inspired, exciton transport via ENAQT, synthesized via in situ and swelling methods, enhanced photocurrent in the swollen system	Bio-inspired photovoltaic assembly	0.59%.	[118]
10	PAA-G-PABD-Fe_3_O_4_NRs Hydrogel	Nanocomposite hydrogel with Fe_3_O_4_ nanorods and [FeSCN]^2+^ complex, enhanced electron transfer, and high thermal stability	Hybrid Solar Cell (FTO|TiO_2_/Gel|Al)	6.08% (2100% improvement over control)	[119]

**Table 4:** Overview of hydrogel materials and their applications in energy devices.

No.	Hydrogel Material	Target Application	Device Type	Ref.
1	Polyacrylamide (PAM)	Supercapacitor	Energy storage	[120]
2	Poly(N-isopropylacrylamide) (PNIPAM)	Solar energy harvesting	Thermo-responsive solar systems	[121]
3	Poly(vinyl alcohol) (PVA)	Solar energy conversion	Photovoltaic cells	[122]
4	Poly(ethylene glycol) (PEG)	Fuel cell applications	Electrochemical systems	[123]
5	Chitosan-based hydrogel	Battery technology	Rechargeable energy systems	[124]
6	Poly(acrylic acid) (PAA)	Fuel cells	Proton exchange membranes	[125]
7	Poly(vinyl pyrrolidone) (PVP)	Solar cells	Photovoltaic devices	[126]
8	Alginate-based hydrogel	Battery systems	Lithium-ion batteries	[127]
9	Poly(ethylene oxide) (PEO)	Thermoelectric conversion	Waste heat recovery devices	[128]
10	Gelatin-based hydrogel	Supercapacitor	Energy storage	[129]
11	Poly(acrylamide-co-sodium acrylate)	Energy harvesting	Triboelectric nanogenerators	[130]
12	Poly(2-hydroxyethyl methacrylate) (PHEMA)	Battery systems	Lithium-ion batteries	[131]
13	Poly(ethylene glycol diacrylate) (PEGDA)	Solar energy	Dye-sensitized solar cells	[132]
14	Poly(N-vinylcaprolactam) (PVNCL)	Thermoelectric devices	Energy harvesting systems	[133]
15	Polythiophene	Supercapacitor	Energy storage	[134]
16	Polystyrenesulfonate	Fuel cells	Proton exchange membranes	[135]
17	Poly(2-isopropyl-2-oxazoline) (PiPOx)	Energy storage	Sodium-ion batteries	[136]
18	Poly(N,N-dimethylacrylamide) (PDMA)	Supercapacitor	Energy storage	[137]
19	Poly(itaconic acid) (PA)	Solar energy	Photovoltaic devices	[138]
20	PANa–PVP–TiO_2_(NH_2_) hybrid hydrogel	High ionic conductivity & flexibility	Quasi-solid-state Zn–air battery	[139]
21	Polyacrylamide (PAM)–Gelatin composite hydrogel	Enhanced ionic conductivity	Zn–air battery	[140]
22	Dual-ion conducting hydrogel (SDICH)—PVA-based with Na^+^ and Mg^2+^ ions	Intelligent biomedical electronics, wound healing	Fully stretchable Mg–air battery	[141]
23	In situ cross-linked polyacrylic acid (PAA)–KOH hydrogel	Flexible and stretchable energy storage	Kirigami-inspired Zn–air battery	[142]
24	Zirconium hydroxide hydrogel	Gas separation and conductivity enhancement	Alkaline water electrolyzer diaphragm	[143]
25	Natural polymer hydrogels (e.g., chitosan, alginate, lignin)	Water purification and environmental remediation	Hydrogel-based filtration systems	[144]
26	Hydrogel photocatalysts (e.g., TiO_2_, g-C_3_N_4_ embedded hydrogels)	Solar-driven water splitting and pollutant degradation	Photocatalytic water electrolyzer	[145]

## Data Availability

No new data were created or analyzed in this study.
